# Baihui‐Penetrating‐Qubin Acupuncture Attenuates Neurological Deficits Through SIRT1/FOXO1 Reducing Oxidative Stress and Neuronal Apoptosis in Intracerebral Hemorrhage Rats

**DOI:** 10.1002/brb3.70095

**Published:** 2024-12-16

**Authors:** Shan‐Shan Dong, Ming‐Yue Li, Xue‐Ping Yu, Yu‐Na Kan, Xiao‐Hong Dai, Lei Zheng, Hong‐tao Cao, Wen‐Hui Duan, En‐Li Luo, Wei Zou

**Affiliations:** ^1^ Acupuncture and Moxibustion Department First Affiliated Hospital of Heilongjiang University of Chinese Medicine Harbin Heilongjiang P. R. China; ^2^ Second Department of Traditional Chinese Medicine, Medical School South China Hospital, Shenzhen University Shenzhen P. R. China; ^3^ Guangdong Key Laboratory for Biomedical Measurements and Ultrasound Imaging, National‐Regional Key Technology Engineering Laboratory for Medical Ultrasound, School of Biomedical Engineering Shenzhen University Medical School Shenzhen China; ^4^ School of Basic Medicine Guizhou University of Traditional Chinese Medicine Guiyang P. R. China

**Keywords:** acupuncture, brain injury, intracerebral hemorrhage (ICH), neuroprotection, neuronal apoptosis, oxidative stress, SIRT1/FOXO1

## Abstract

**Background:**

Intracerebral hemorrhage (ICH) is a significant global disease with high mortality and disability. As of now, there is no effective therapy available. Oxidative stress and neuronal apoptosis play essential roles in ICH, determining neuronal survival. In our preliminary studies, we found that Baihui‐penetrating‐Qubin acupuncture could improve neurological deficits and neuropathological damage in the perihematomal area in ICH rats. The SIRT1/FOXO1 signaling pathway has been reported to mediate antioxidant and anti‐neuronal apoptosis. This study aimed to investigate the effects of Baihui‐penetrating‐Qubin acupuncture on oxidative stress and neuronal apoptosis after ICH and the role of SIRT1/FOXO1 in acupuncture's neuroprotection.

**Methods:**

ICH rat models were established by autologous tail blood (50 µL) infusion into the caudate nucleus. EX527, SIRT1‐specific inhibitor was intraperitoneally administered 3 days before ICH. Baihui‐penetrating‐Qubin acupuncture treatment was performed once a day for 30 min after ICH. Neurological deficits were evaluated using the modified neurological severity score (mNSS). Brain edema was evaluated using brain water content. HE staining and Nissl staining were used to evaluate neuropathological damage in the perihematomal area. Terminal deoxynucleotidyl transferase dUTP nick end labeling was used to quantify neuronal apoptosis. Specific kits were used to detect the levels of SOD, CAT, GSH‐Px in the brain. The oxidative DNA damage was evaluated using enzyme‐linked immunosorbent assay to detect the level of 8‐hydroxyguanosine (8‐OHdG). Western blot was used to evaluate the expressions of SIRT1, Ac‐FOXO1, FOXO1, Bcl‐2, and Bax. Immunofluorescence staining was conducted to detect the cellular localization of SIRT1.

**Results:**

Baihui‐penetrating‐Qubin acupuncture improved the neurological deficits and brain edema, reduced the pathological injury and neuronal degeneration in 3 days in the perihematomal area after ICH. Mechanistically, acupuncture reduced oxidative stress injury and neuronal apoptosis via activating SIRT1/FOXO1 pathway. The neuroprotective effects of acupuncture were abolished by injection of the SIRT1 inhibitor EX527.

**Conclusions:**

Baihui‐penetrating‐Qubin acupuncture could reduce oxidative stress and neuronal apoptosis, at least in part, through the SIRT1/FOXO1 signaling pathway, improving neurological deficits and neuropathological damage after ICH. These findings suggest that Baihui‐penetrating‐Qubin acupuncture is an effective therapy for ICH, as well as targeting SIRT1 signaling to promote neuron survival could be a potential therapeutic strategy.

## Graphical abstract

Baihui‐penetrating‐Qubin acupuncture attenuates oxidative stress and neuronal apoptosis in intracerebral hemorrhage rats through SIRT1/FOXO1 signaling pathway. Baihui‐penetrating‐Qubin acupuncture activates SIRT1, inhibits FOXO1 acetylation, and promotes FOXO1 accumulation. This accumulation upregulates the expression of antioxidant enzymes, including SOD, CAT, and GSH‐Px, and downregulates the expression of oxidative damage products such as MDA and 8‐OHdG, further attenuating neuronal apoptosis against brain injury after ICH.

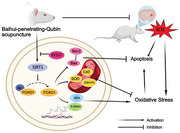

## 1 Introduction

Intracerebral hemorrhage (ICH) is a significant global disease caused by the rupture of cerebral blood vessels and the blood entries into the brain parenchyma, affecting approximately 2 million people worldwide every year (Cordonnier et al. 2018). According to statistics, the annual incidence rate of ICH is (10–30)/100,000 (Li et al. 2023). Only 50% of patients are able to survive for 1 year after ICH, and those who do survive often experience severe neurological deficits as sequelae (Puy et al. 2023). Between 1990 and 2016, ICH caused 2.8 million deaths and 64.5 million disability‐adjusted life years (Sporns et al. 2021). Until now, the global incidence of ICH has been a continuous increase (Puy et al. 2023). Oxidative stress and neuronal apoptosis play a crucial role in secondary brain injury after ICH, importantly impacting neuronal survival (Feng et al. 2023). Once blood enters the brain parenchyma after ICH, it can induce oxidative stress through various mechanisms, including cytotoxicity, excitotoxicity, and mitochondrial dysfunction, leading to oxidation of cellular macromolecules and ultimately resulting in neuronal apoptosis and damage in the perihematomal area (Duan et al. 2016). Targeting to reduce oxidative stress and neuronal apoptosis could decrease the mortality and disability associated with ICH in animals and patients (Liao et al. 2017; Xiao et al. 2022). Therefore, suppressing oxidative stress and neuronal apoptosis is an effective approach to regulate the secondary brain injury after ICH.

Silent information regulator 1 (SIRT1), which belongs to the histone deacetylase (Sirtuin) family, plays a crucial role in neuronal survival (Cai et al. 2016). SIRT1 is a nutrient‐ and energy‐sensitive signaling factor with diverse biological functions, including transcription, genome maintenance, metabolism regulation, and antioxidant response (Zhao and Zhou 2020). SIRT1 exerts its biological activity by targeting histones and transcription factors for deacetylation, meanwhile the forkhead box transcription factor 1 (FOXO1) is one of the downstream targets of SIRT1 (Jalgaonkar et al. 2022). FOXO1 acts as a sensing element in antioxidant signaling pathways and regulates oxidative stress injury and neuronal apoptosis (Hu et al. 2019). Notably, the SIRT1/FOXO1 pathway is a core cellular mechanism in nervous system diseases, promoting neuronal survival, reducing toxic substance accumulation, maintaining mitochondrial homeostasis, and facilitating nervous system repair (Maiese 2021). Therefore, exploring the effects of the SIRT1/FOXO1 pathway on oxidative stress and neuronal apoptosis after ICH is of great significance.

Acupuncture is a historical practice of traditional Chinese medicine, which has been endorsed by the World Health Organization (WHO) as an alternative or complementary approach for stroke treatment (Zhou et al. 2019). In this study, we utilized a specific acupuncture method called Baihui‐penetrating‐Qubin, which involves inserting a single needle through Baihui (GV20) to Qubin (GB7) acupoints, allowing for simultaneously stimulating three brain regions of the frontal, parietal, and temporal lobes. In our preliminary studies (Guan et al. 2021; Li et al. 2020, 2021, 2018;P. Liu et al. 2021; Zhang et al. 2018; Zou et al. 2015), we found that Baihui‐penetrating‐Qubin acupuncture is an effective therapy for improving neurological deficits after ICH. Its protective effects are related to the regulation of multiple mechanisms of neuroinflammation, apoptosis, autophagy, ferroptosis, nerve regeneration, etc., especially in improving the microenvironment of lesions and promoting neuronal survival. For example, acupuncture inhibited apoptosis by enhancing mitophagy (Guan et al. 2021), activated the p62‐Keap1‐Nrf2 signaling pathway to regulate ferroptosis (Li et al. 2021), and upregulated the expression of neurotrophic factors of Nestin, GDNF, and bFGF in the perihematomal area (Li et al. 2020). However, it is still unclear whether the neuroprotection of acupuncture is related to the SIRT1/FOXO1 pathway. In this study, we investigated the effects of Baihui‐penetrating‐Qubin acupuncture on neurological deficits, brain edema, neuropathological damage, oxidative stress injury, neuronal apoptosis, and the expressions of SIRT1/FOXO1. Additionally, we explored the associated mechanism using a SIRT1‐specific inhibitor.

## 2 Materials and Methods

### 2.1 Animals

Adult male Sprague‐Dawley rats weighing 280–320 g were sourced from the Center of Drug Safety and Evaluation, Heilongjiang University of Chinese Medicine, China(license No. SCXK (hei) 2018‐007). The rats were housed in a controlled environment, maintained at a temperature of 20–23°C, humidity of 50–55%, and a 12:12‐hour light–dark cycle. They were provided with ad libitum access to food and water. All experimental protocols involving the care of the rats were approved by the Ethics Committee on Experimental Animals of the Heilongjiang University of Chinese Medicine (Approval No. 2019‐10‐02‐01).

### 2.2 Experimental Design

**FIGURE 1:**
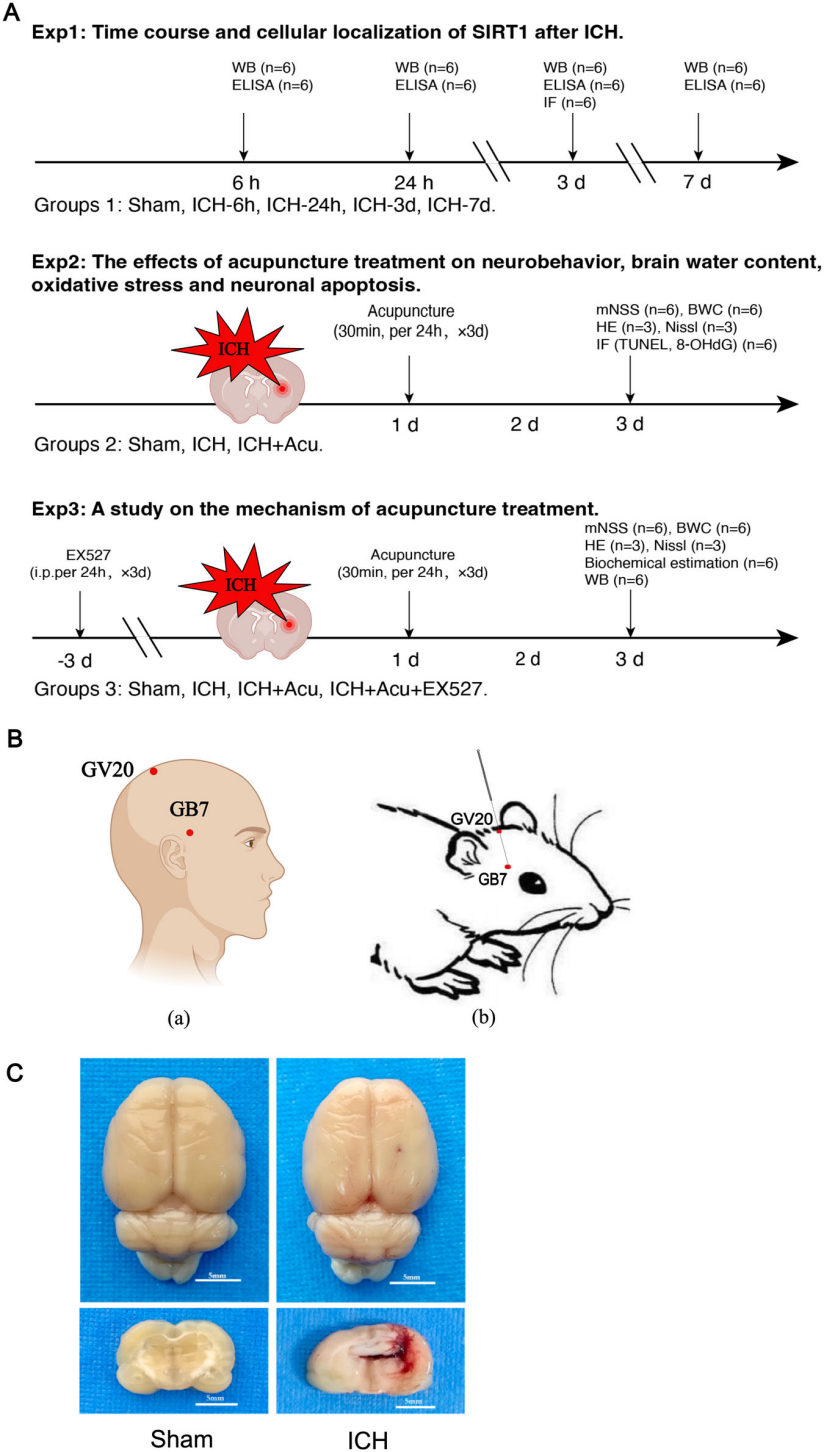
Description of experimental setup, Baihui‐penetrating‐Qubin acupuncture technique, and ICH rat models. (A) Experimental design and animal groups. Exp: experiment; ICH: intracerebral hemorrhage; WB: western blot; ELISA: enzyme‐linked immunosorbent assay; IF: immunofluorescence; mNSS: modified neurological severity score; BWC: brain water content; H&E: hematoxylin and eosin staining; TUNEL: terminal deoxynucleotidyl transferase‐ mediated dUTP nick end labeling; i.p.: intraperitoneal injection; mNSS: modified neurological severity score; 8‐OHdG: 8‐hydroxyguanosine. (B) The illustration of Baihui‐penetrating‐Qubin acupuncture treatment. (a) Location of GV20 and GB7 in human. (b) Schematic diagram of Baihui‐penetrating‐Qubin acupuncture in a rat. Baihui (GV20) is located at the intersection of the sagittal midline and the line between the two ears; Qubin (GB7) is located in the posterior 2/3 of the line connecting the right orbital margin and porus acusticus externus.(C)Location of the ICH hematoma. Representative images of coronal brain sections from the Sham and ICH groups, showing the site of blood transfusion within the caudate putamen region.

As represented in Figure 1A, a total of three distinct experiments were conducted.

In Experiment 1, we aimed to characterize the time course of expressions of endogenous SIRT1 and 8‐hydroxyguanosine (8‐OHdG), as well as the cellular localization of SIRT1 in the perihematomal area after ICH. A total of 30 rats were randomly assigned to five groups (*n* = 6 per group): Sham, ICH‐6 h, ICH‐24 h, ICH‐3d, and ICH‐7d. Western blotting and enzyme‐linked immunosorbent assay (ELISA) were performed at 3 days after ICH. Additionally, immunofluorescence staining was conducted on a separate set of six rats (*n* = 3 per group) from the Sham and ICH‐3d groups to detect the cellular localization of SIRT1. Based on the expression of SIRT1, we chose the timing for acupuncture studies.

In Experiment 2, we aimed to evaluate the neuroprotective effects of acupuncture. A total of 36 rats were randomly assigned to three groups (*n* = 12 per group): Sham, ICH, and ICH+Acu (ICH+Acupuncture). All groups underwent neurological deficits evaluation, brain water content measurement, hematoxylin and eosin (H&E) staining, terminal deoxynucleotidyl transferase dUTP nick end labeling (TUNEL) staining, and double immunofluorescence staining at 3 days after ICH.

In Experiment 3, we aimed to explore the neuroprotective mechanism of acupuncture. A total of 48 rats were randomly assigned to four groups (*n* = 12 per group): Sham, ICH, ICH+Acu, and ICH+Acu+EX527. Similar to Experiment 2, all groups underwent neurological deficits evaluation, brain water content measurement, H&E staining, TUNEL staining, biochemical estimation, and Western blotting at 3 days after ICH.

### 2.3 ICH Models

The autologous blood model of ICH was conducted following previously established methods (Liu et al. 2018, 2021). Rats were anesthetized and securely positioned in a prone position on a stereotaxic frame (STW‐3X; Chengdu Instrument Factory; Chengdu; China). To fully expose the bregma and coronal sulcus, a 1‐cm incision was made along the midline of the scalp. An injection site was marked, and a 1‐mm hole was drilled through the designated point to reach the surface of the dura mater using a dental drill. Autologous tail blood (50 µL) was then slowly injected into the right caudate nucleus through the hole using a 100 µL microsyringe (Hamilton Company, Reno, NV, USA) at a rate of 20 µL/min. The injection coordinates relative to the bregma were as follows: 3.5 mm lateral, 0.2 mm posterior, and 6 mm beneath the horizontal plane of the brain surface (Figure 1C). The microsyringe was gently removed 5 min after the injection. The incision was sealed with dental cement, followed by suturing, bandaging, and sterilization. The success of ICH models was determined based on the Berderson's scale (Bederson et al. 1986), whereby rat scoring 1–3 was considered to have a successful ICH model.

### 2.4 Drug Administration

The SIRT1‐specific inhibitor, EX527 (ab141506, Abcam), was administered to the rats in ICH+Acu+EX527 group via intraperitoneal injection, with a dosage of 10 mg/kg (X. Zhang et al. 2020). This administration was performed once daily for a period of 3 days preceding the ICH operation.

### 2.5 Acupuncture Treatment

Acupuncture treatment was administered to the rats in ICH+Acu and ICH+Acu+EX527 groups. Approximately 3 h after establishing the ICH models, when the rats were fully awake, they were placed in a prone position on boards to begin acupuncture treatment. A sterile Andy acupuncture needle (diameter: 0.35 mm, length: 40 mm, Suzhou Medical Instrument Co., Ltd, Suzhou, China) was inserted obliquely through “Baihui” (GV20, which is situated at the intersection of the sagittal midline and the line between the two ears) to “Qubin” (GB7, acupoint located on the lesion side), with a 15 mm depth (Figure 1B). This treatment was administered once every 24 h, with each session lasting 30 min, over a span of 3 days. During needle retention, the needle was alternately rotated manually along clockwise and counter‐clockwise directions at a speed of 200 turns/min. Stimulation lasted three times, for 5 min each, with 5‐minute intervals between stimulations. No anesthesia was required during the entire acupuncture treatment for the rats.

### 2.6 Neurological Deficits Evaluation

Neurological deficits were assessed 3 days after ICH using the 18‐point modified neurological severity score (mNSS), as previously described (Xia et al. 2020). The evaluation was conducted by two trained researchers who were blinded to the grouping of the animals. The mNSS includes assessments of motor function, sensory function, balance, and reflexes. Neurological function is scored on a scale of 0 to 18 points, where 0 indicates normal performance and 18 indicates severe neurological deficit (1−6, mild injury; 7−12, moderate injury; 13−18, severe injury) (Xia et al. 2020). Higher scores indicate more severe neurological impairment.

### 2.7 Brain Water Content

Brain water content (BWC) was measured at 3 days after ICH, as previously described (Lee et al. 2008). After anesthesia, rats were decapitated and the wet weights of brain samples were immediately measured. Then the samples were dried at 100°C for 24 h to obtain the dry weight. The BWC was calculated as [(wet weight–dry weight)/wet weight] × 100%.

### 2.8 Histological Analysis

Rats were deeply anesthetized and transcardially perfused with physiological saline solution, followed by fixation in 4% paraformaldehyde (PFA), as previously described (Zuo et al. 2019). Brain samples, approximately 4 mm thick, were collected around the site of blood injection and fixed in 4% PFA containing 1% diethyl pyrocarbonate at 4°C for 24 h. The brain samples were embedded in paraffin, followed by xylene treatment for transparency and dehydration using a gradient concentration of alcohol. Brain sections (5 µm thick) were obtained using a microtome (RM2016, Leica Biosystems, China) and prepared for immunohistochemistry, double immunofluorescence, TUNEL staining, H&E staining, and Nissl staining.

### 2.9 Protein and Biochemical Analysis

Rats were deeply anesthetized and decapitated. Brain samples, approximately 4 mm thick (centered around the injection site, extending 2 mm anterior and posterior), were collected and immediately stored in liquid nitrogen, then preserved at −80°C. These samples were used for subsequent Western Blot, ELISA, and biochemical estimation.

### 2.10 Immunofluorescence Staining

Brain sections (5 µm thick) were first blocked with 1% BSA for 1 h. Subsequently, the sections were incubated overnight at 4°C with primary antibodies, including 8‐OHdG (Bioss, bs‐1278R, 1:100), SIRT1 (Abcam, ab189494, 1:100), and NeuN (Proteintech, 66836‐1‐Ig, 1:100). Afterwards, the sections were incubated with the appropriate fluorophore secondary antibodies (Thermo Fisher Scientific, Invitrogen, A‐11029 for Alexa Fluor 488 and A‐11037 for Alexa Fluor 594, 1:250) for 1 h at room temperature. Cell nuclei were counterstained with 4′,6‐diamidino‐2‐phenylindole (DAPI, D9542, Sigma). Fluorescent images were captured using a fluorescence microscope (Nikon, Tokyo, Japan, Nikon Eclipse Ti‐SR). The fluorescence intensity of positive cells in three non‐overlapping fields (400× magnification) was quantified using the ImageJ software.

### 2.11 TUNEL Staining

Double staining of the neuron marker NeuN and TUNEL was performed using the in situ Apoptosis Detection Kit (Roche, Basel, Switzerland, 12156792910), following the manufacturer's instructions. The percentage of TUNEL‐positive neurons was calculated as the ratio of TUNEL‐positive neurons to the total number of neurons, multiplied by 100.

### 2.12 HE Staining

Paraffin sections were subjected to HE staining using a standard protocol. The organizational structure in the perihematomal area was observed under a microscope (Nikon, Nikon Eclipse E100, Tokyo, Japan) at a magnification of 200× and 400×.

### 2.13 Nissl Staining

Nissl staining was performed to assess NeuN injury, following previously established methods (Chen et al. 2018). Briefly, brain sections were deparaffinized with xylene, rehydrated through a series of graded alcohol solutions, and stained with Cresyl violet solution to visualize degenerating neurons in the perihaemorrhagic area. Images were captured using an optical microscope (Nikon, Nikon Eclipse E100, Tokyo, Japan) at a magnification of 200× and 400×.

### 2.14 Biochemical Estimation

After anesthesia, brain samples were rapidly removed from the rats. The levels of antioxidant enzymes of superoxide dismutase (SOD), catalase (CAT), glutathione peroxidase (GSH‐Px), and the oxidative damage product malondialdehyde (MDA), were detected using commercially available kits (Nanjing Jiancheng Bioengineering Institute) following the manufacturer's instructions. The specific kits used were as follows: SOD activity assay kit (No. A001‐3‐2), CAT activity assay kit (No. A007‐1‐1), GSH‐Px activity assay kit (No. A005‐1‐2), and MDA activity assay kit (No. A003‐1‐2).

### 2.15 Enzyme‐Linked Immunosorbent Assay (ELISA)

After anesthesia, brain samples were rapidly removed from the rats. The level of 8‐OHdG in brain homogenates was measured using an ELISA assay (MEIMIAN, MM‐0224R1 96T) following the manufacturer's instructions.

### 2.16 Western Blotting Analysis

Western blotting was conducted at 3 days after ICH, following a previously described protocol. Briefly, rats were anesthetized and decapitated, and the corresponding protein was extracted. Equal amounts of protein (40 µg) were loaded onto SDS‐PAGE gels, electrophoresed, and transferred to nitrocellulose membranes. The membranes were then blocked and incubated overnight at 4°C with the following primary antibodies: SIRT1 (Abcam, ab189494, 1:1000), FOXO1 (Abcam, ab179450, 1:1000), Ac‐FOXO1 (Affinity, AF2305, 1:1000), Bcl‐2 (Bioss, bsm‐33047 M, 1:1000), Bax (Abcam, ab32503, 1:1000), and β‐actin (Abcam, ab227387, 1:5000). The next day, the membranes were incubated with the appropriate secondary antibody at room temperature for 2 h. The immunoblots were visualized using the ECL Plus chemiluminescence reagent kit (BQD, Ba1059). The optical density of the blot bands was quantified using ImageJ software.

### 2.17 Statistical Analysis

All data were analyzed using GraphPad Prism 8.4.0 (GraphPad Software, LLC) and presented as the mean ± standard deviation (SD). The Shapiro‐Wilk test was used to assess the normality of the distribution. Differences among groups were compared using one‐way ANOVA with Bonferroni post hoc test. A simple linear regression analysis was used to determine the relationship between the expressions of SIRT1 and 8‐OHdG. A *p*‐value < 0.05 was considered statistically significant.

## 3 Results

### 3.1 The Expressions of Endogenous SIRT1 and 8‐OHdG in the Perihematomal Area After ICH

**FIGURE 2:**
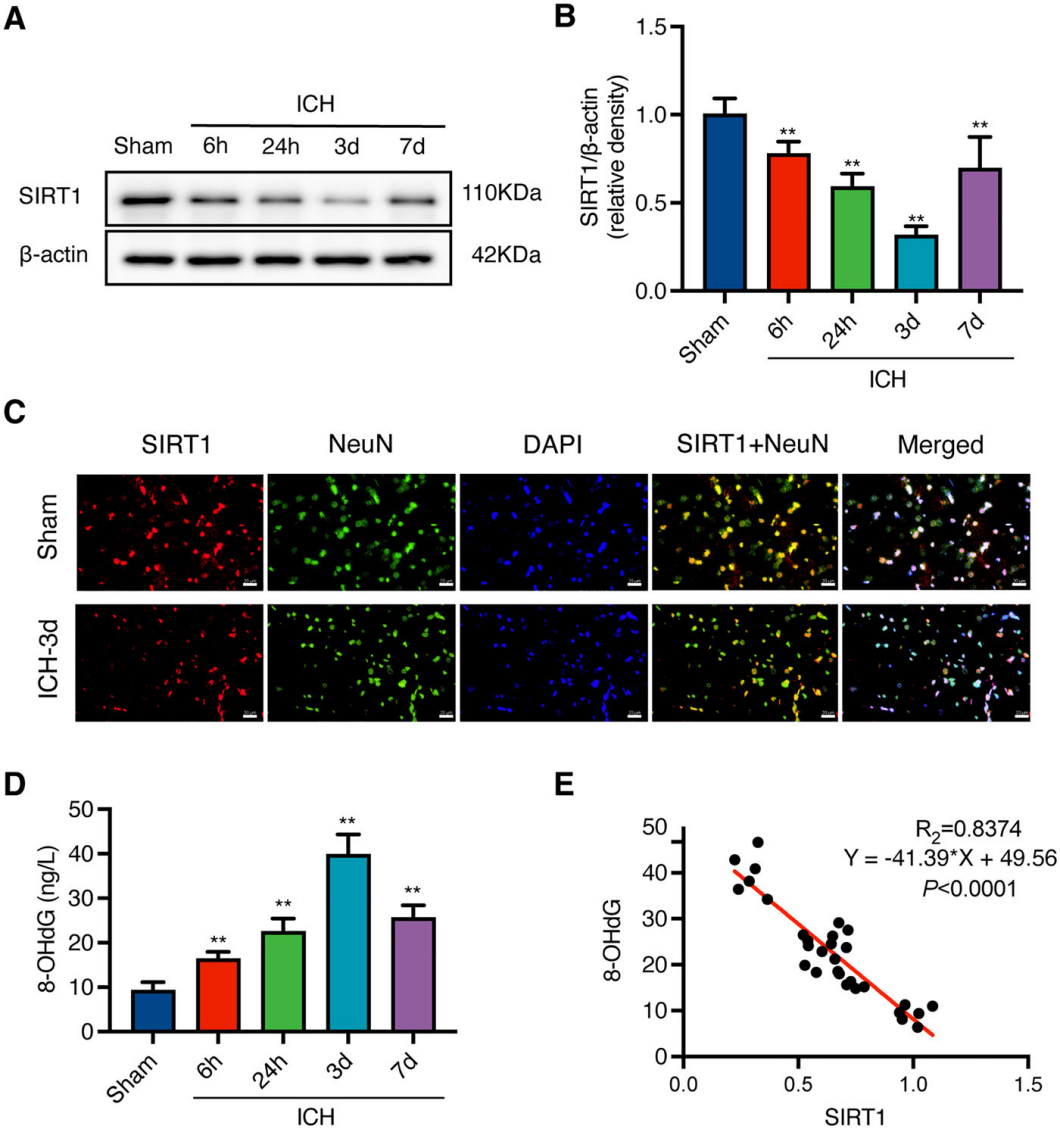
Time course of SIRT1 and 8‐OHdG expression in the perihematomal area after ICH. (A and B) Representative Western blot bands and quantitative analysis of SIRT1 expression levels in the perihematomal area after ICH. ICH: intracerebral hemorrhage; SIRT1: silent information regulator 1. (C) Representative micrographs of SIRT1 (red) /NeuN (green) co‐immunofluorescence staining in the perihematomal area after ICH (400×, *n* = 3). DAPI: diamidino phenylindole. (D) Time course of 8‐OHdG expression in the perihematomal area after ICH. Data are presented as the mean ± SD(*n* = 6); *p < 0.05, **p < 0.01 vs Sham group. 8‐OHdG: 8‐hydroxyguanosine. (E) Correlation analysis of SIRT1 and 8‐OHdG expression levels in the perihematomal area after ICH.

The time course of SIRT1 expression in the perihematomal area was assessed using Western blotting. The results demonstrated that, compared to the Sham group, SIRT1 expression levels decreased from 6 h to 7 days and reached their lowest levels at 3 days after ICH (*F* = 104.40, **p < 0.01, Figure 2A,B). Based on these findings, the 3‐day time point after ICH was selected as the optimal time window for subsequent experiments.

To confirm the distribution of SIRT1 in neurons after ICH, double immunofluorescence staining of SIRT1 with NeuN (a neuron marker) was performed. The results showed that SIRT1 was highly expressed in neurons in rats. Furthermore, compared to the Sham group, the number of SIRT1‐positive neurons was reduced in the ICH‐3d group (Figure 2C).

The time course of 8‐OHdG expression was assessed using an ELISA assay. The results revealed that, compared to the Sham group, the expression of 8‐OHdG began to increase as early as 6 h after ICH and peaked at 3 days (*F* = 93.37, **p < 0.01, Figure 2D). There was a negative correlation between the expressions of SIRT1 and 8‐OHdG in the perihematomal area after ICH (Figure 2E).

### 3.2 Acupuncture Improved the Neurological Deficits and Brain Edema After ICH

**FIGURE 3:**
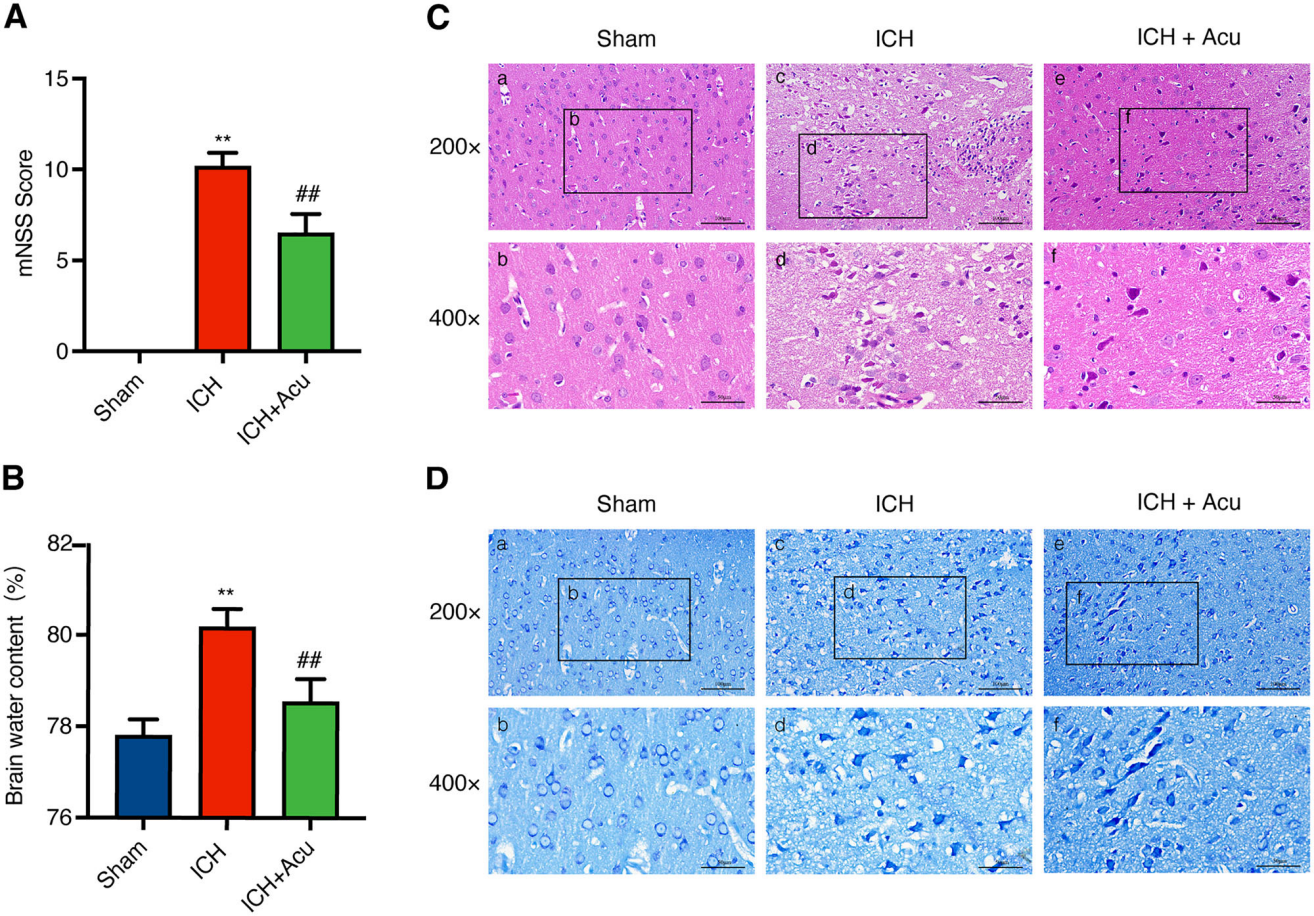
Effects of Baihui‐penetrating‐Qubin acupuncture on the neurobehavioral deficits and neuropathological damage at 3 days after ICH. (A) The mNSS score after ICH (n = 6). mNSS: modified neurological severity score; ICH+Acu: intracerebral hemorrhage + Acupuncture.(B) Quantification of brain water content after ICH (n = 6). Data are presented as the mean ± SD; *p < 0.05, **p < 0.01 vs Sham group;^#^ *p* < 0.05, ^##^ *p* < 0.01 vs ICH group. (C and D) H&E staining and Nissl staining in the perihematomal area after ICH (200×, 400×, *n* = 3). The pictures of a, c, e represent the H&E and Nissl staining of tissue around the hematoma. The b, d, and f insets are magnifications of a, c, and e, respectively. Scale bars, 200×: 100 µm; 400×: 50 µm. mNSS: Modified neurological severity score; H&E: hematoxylin and eosin staining.

The neurological deficits were assessed using the mNSS at 3 days after ICH. The results indicated that there were significant neurobehavioral deficits in the ICH group compared to the Sham group (*F* = 286.30, ^**^ *p* < 0.01, Figure 3A). However, GV20‐GB7 acupuncture treatment significantly improved the neurobehavioral performance in the mNSS test after ICH when compared to the ICH group (^##^ *p* < 0.01, Figure 3A).

Brain edema was evaluated by estimating the BWC at 3 days after ICH. The findings revealed that the BWC in the ICH group significantly increased compared to the Sham group (*F* = 81.34, ^**^ *p* < 0.01, Figure 3B). Interestingly, acupuncture treatment effectively improved BWC when compared to the ICH group (^##^ *p* < 0.01, Figure 3B). These results suggest that acupuncture may improve both neurological deficits and brain edema after ICH.

### 3.3 Acupuncture Reduced Pathological Injury and Neuronal Degeneration in the Perihematomal Area After ICH

H&E staining was used to observe the pathological injury in the perihematomal area at 3 days after ICH. The results demonstrated that the brain structure in the Sham group exhibited tight and uniform characteristics (Figure 3C). In contrast, the ICH group showed severe disruption of the brain tissue, with a substantial presence of necrotic cells, inflammatory cells, and interstitial edema (Figure 3C). The ICH+Acu group exhibited a slightly clearer brain tissue with a reduced amount of necrotic and inflammatory cells, as well as mild tissue edema when compared to the ICH group (Figure 3C).

Nissl staining was used to evaluate neuronal damage in the perihematomal area at 3 days after ICH. The results demonstrated that the neuronal structure in the Sham group remained intact, regular, and rich in Nissl bodies (Figure 3D). Conversely, the ICH group exhibited disarranged and lost neurons, with pyknosis of neuronal bodies and the disappearance of nuclei (Figure 3D). Furthermore, acupuncture treatment significantly reduced neuronal damage (Figure 3D).

### 3.4 Acupuncture Reduced Neuronal Apoptosis in the Perihematomal Area After ICH

**FIGURE 4:**
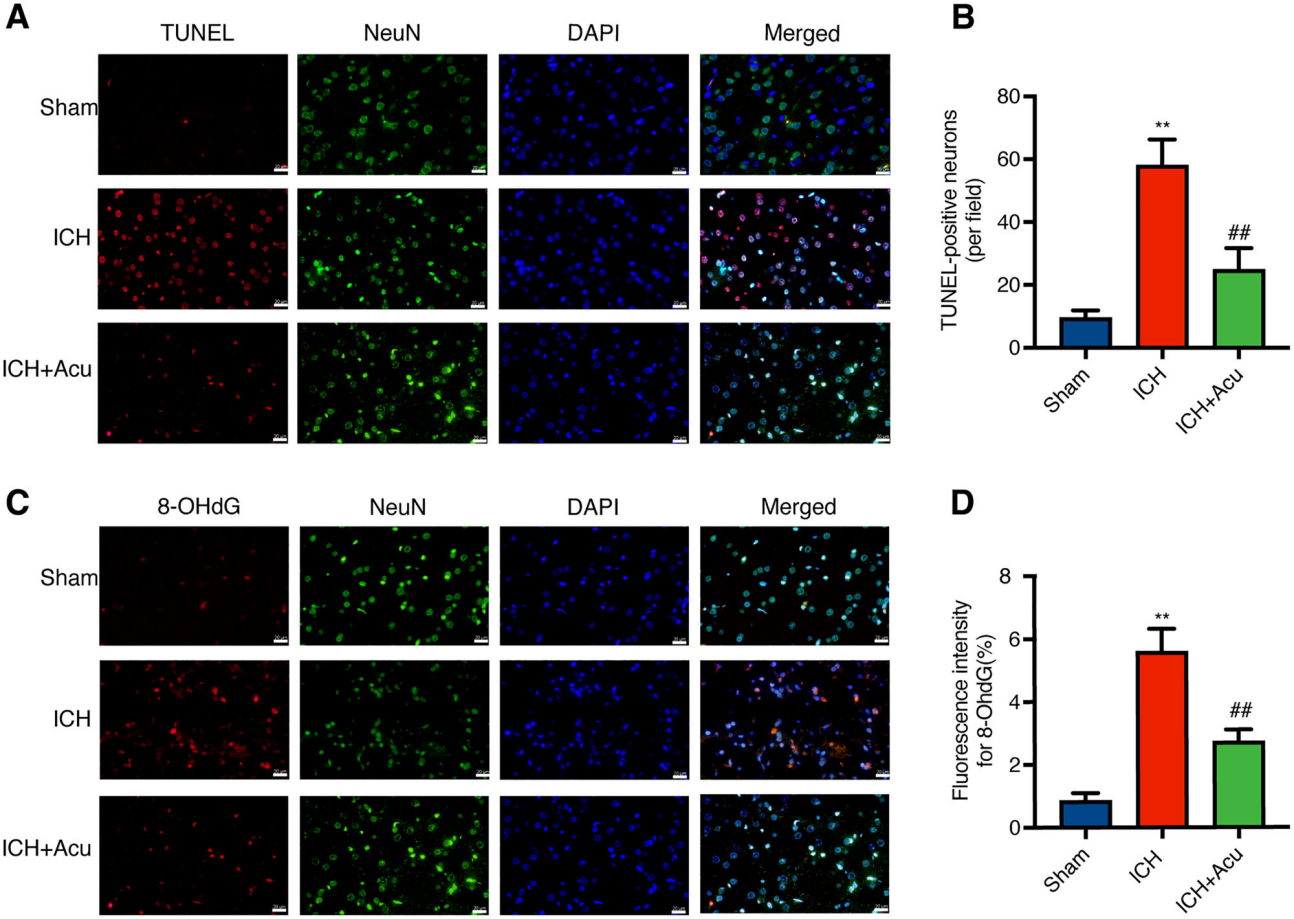
Effects of Baihui‐penetrating‐Qubin acupuncture on neuronal apoptosis and oxidative stress in the perihematomal area at 3 days after ICH. (A and B) Representative micrographs and quantitative analysis of TUNEL‐positive neurons in the perihematomal area after ICH (400×). TUNEL: terminal deoxynucleotidyl transferase‐mediated dUTP nick end labeling; DAPI: diamidino phenylindole; ICH+Acu: intracerebral hemorrhage.(C and D) Representative micrographs and quantitative analysis of 8‐OHdG (red)/NeuN (green) co‐immunofluorescence staining in the perihematomal area after ICH (400×). Scale bars: 50 µm. Data are presented as the mean ± SD (*n* = 6); *p < 0.05, **p < 0.01 vs Sham group; ^#^ *p* < 0.05, ^##^ *p* < 0.01 vs ICH group. 8‐OHdG: 8‐hydroxyguanosine.

TUNEL staining was performed to evaluate neuronal apoptosis in the perihematomal area at 3 days after ICH. The ICH group exhibited a significant increase in the number of TUNEL‐positive neurons compared to the Sham group (*F* = 90.33, ^**^ *p* < 0.01, Figure 4A,B). Acupuncture treatment significantly reduced the number of neuronal apoptosis compared to the ICH group (^##^ *p* < 0.01, Figure 4A,B).

### 3.5 Acupuncture Reduced Oxidative Stress Injury in the Perihematomal Area After ICH

Double immunofluorescence staining of 8‐OHdG (a marker of oxidative DNA damage) with NeuN was performed to investigate the effects of acupuncture on oxidative stress injury in the perihematomal area at 3 days after ICH. The results revealed a significant increase in the fluorescence intensity of 8‐OHdG in the ICH group compared to the Sham group (*F* = 139.60, **p < 0.01, Figure 4C,D). Acupuncture treatment significantly decreased the fluorescence intensity of 8‐OHdG compared to the ICH group (^##^ *p* < 0.01, Figure 4C,D).

### 3.6 Acupuncture Reduced Oxidative Stress and Neuronal Apoptosis via the SIRT1/FOXO1 Pathway

**FIGURE 5:**
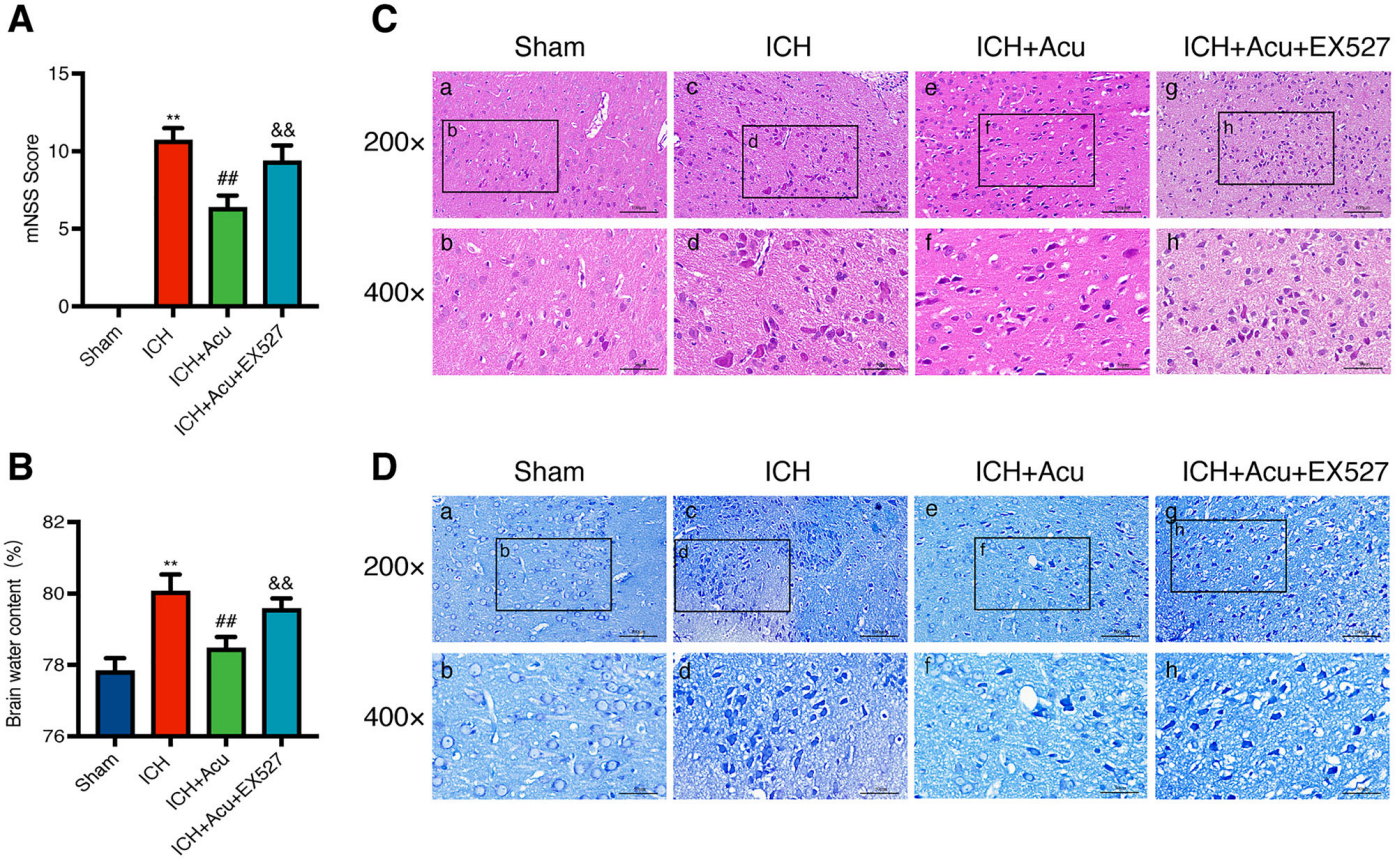
Effects of Baihui‐penetrating‐Qubin acupuncture on neurobehavioral deficits and neuropathological damage after ICH with pre‐injection of EX527. (A) The mNSS score after ICH (*n* = 6). (B) The brain water content after ICH (*n* = 6). Data are presented as the mean ± SD; *p < 0.05, **p < 0.01 VS. Sham group; ^#^ *p* < 0.05, ^##^ *p* < 0.01 VS. ICH group; ^&^ *p* < 0.05, ^&&^ *p* < 0.01 VS. ICH + Acu group. (C–D) H&E and Nissl staining in the perihematomal area after ICH (200×, 400×, *n* = 3). The pictures of a, c, e, and g represent the H&E and Nissl staining of tissue around the hematoma. The b, d, f, and h insets are magnifications of a, c, e, and g, respectively. Scale bars: 200×: 100 µm; 400×:50 µm. mNSS: Modified neurological severity score; H&E: hematoxylin and eosin staining.

The mNSS results demonstrated that acupuncture treatment significantly improved neurological deficits compared to the ICH group, with lower scores in the ICH+Acu group (*F* = 225.50, ^##^ *p* < 0.01, Figure 5A). This finding is consistent with the results of Experiment 2. Furthermore, when SIRT1 was specifically blocked by EX527, the neurobehavioral deficits were significantly exacerbated in the ICH+Acu+EX527 group compared to the ICH+Acu group (^&&^ *p* < 0.01, Figure 5A).

BWC results revealed that the ICH+Acu group had a significantly improved brain edema compared to the ICH group (*F* = 72.12, ^##^ *p* < 0.01, Figure 5B), which is also consistent with the results from Experiment 2. Additionally, the BWC was further increased in the ICH+Acu+EX527 group compared to the ICH+Acu group (^&&^ *p* < 0.01, Figure 5B).

H&E staining and Nissl staining results showed that the injection with EX527 blocked the protective effects of acupuncture (Figure 5C,D). The ICH+Acu+EX527 group exhibited severe brain damage with increased necrotic cells, inflammatory cell infiltration, and more severe interstitial edema and vacuolization compared to the ICH+Acu group (Figure 5C). Moreover, the pyknosis and degeneration of neuron, as well as the Nissl body, disappeared in the ICH+Acu+EX527 group compared to the ICH+Acu group (Figure 5D).

**FIGURE 6:**
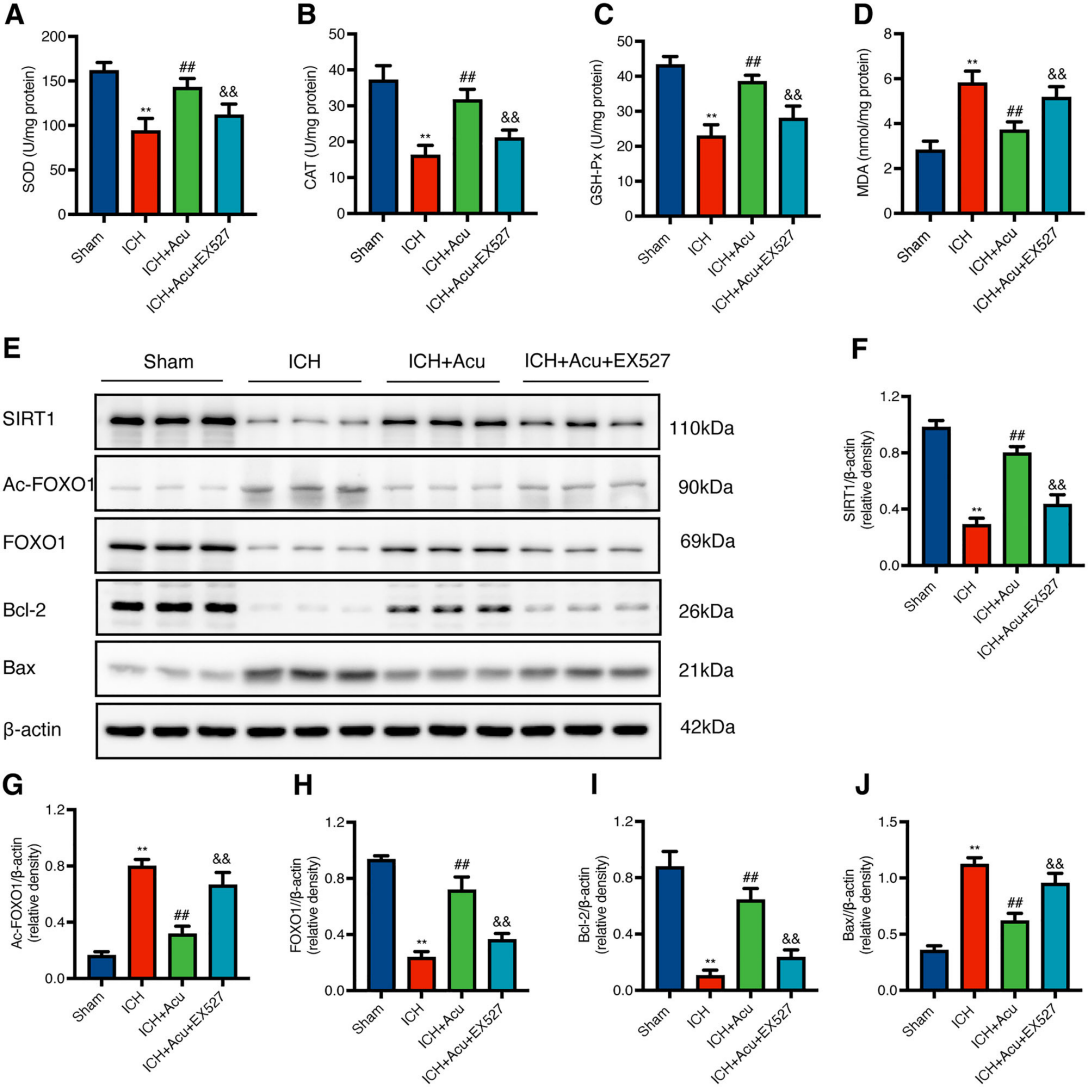
Effects of Baihui‐penetrating‐Qubin acupuncture on the SIRT1‐FOXO1 signaling pathway and expressions of downstream signaling molecules in the perihematomal area at 3 days after ICH. (A‐F) Representative Western blot bands and quantitative analysis of SIRT1, Ac‐FOXO1, FOXO1, Bcl‐2, and Bax in the perihematomal area after ICH. (G‐J) SOD, CAT, GSH‐Px, and MDA levels in the perihematomal area after ICH. Data are presented as the mean ± SD (*n* = 6); *p < 0.05, **p < 0.01 vs Sham group; ^#^ *p* < 0.05, ^##^ *p* < 0.01 vs ICH group; ^&^ *p* < 0.05, ^&&^ *p* < 0.01 vs ICH + Acupuncture group. CAT: catalase; GSH‐Px: glutathione peroxidase; ICH: intracerebral hemorrhage; MDA: malondialdehyde; SOD: superoxide dismutase.

To evaluate the state of oxidative stress in the perihematomal area at 3 days after ICH, the levels of SOD, CAT, GSH‐Px, and MDA were determined. The data revealed that ICH resulted in significant increase in MDA and decrease in SOD, GSH‐Px, and CAT, compared to the Sham group [*F*(SOD) = 35.76, *F*(CAT) = 52.63, *F*(GSH‐Px) = 53.18, *F*(MDA) = 47.45, ^**^ *p* < 0.01, Figure 6A–D]. Acupuncture treatment in the ICH+Acu group alleviated oxidative damage and increased the antioxidant enzymes levels (^##^ *p* < 0.01, Figure 6A–D). Additionally, SIRT1 was specifically blocked by EX527 injection in the ICH+Acu+EX527 group, which significantly suppressed the levels of SOD, GSH‐Px, and CAT while increasing the level of MDA compared to the ICH+Acu group (^&&^ *p* < 0.01, Figure 6A–D).

Western blots were performed to evaluate the activation of SIRT1 and its downstream apoptotic‐related signaling molecules at 3 days after ICH. The results showed that, compared to the Sham group, the ICH group exhibited significantly decreased expressions of SIRT1, FOXO1, and Bcl‐2, whereas the expressions of Ac‐FOXO1 and Bax were remarkably increased [*F*(SIRT1) = 181.10, *F*(FOXO1) = 172.80, *F*(Bcl‐2) = 129.30, *F*(Ac‐FOXO1) = 151.30, *F*(Bax) = 122.90, **p < 0.01, Figure 6E–J]. Furthermore, acupuncture treatment increased the expressions of SIRT1, FOXO1, and Bcl‐2, while decreasing the expressions of Ac‐FOXO1 and Bax in the ICH+Acu group compared to the ICH group (^##^ *p* < 0.01, Figure 6E–J). In contrast, in the ICH+Acu+EX527 group, where SIRT1 was specifically blocked by EX527, the downstream protein expressions of FOXO1 and Bcl‐2 significantly decreased. Additionally, significant overexpression of Ac‐FOXO1 and Bax were observed in the ICH+Acu+EX527 group compared to the ICH+Acu group (^&&^ *p* < 0.01, Figure 6E–J).

## 4 Discussion

In this study, we investigated the neuroprotection of Baihui‐penetrating‐Qubin acupuncture on ICH and its relationship with the SIRT1/FOXO1 mediated antioxidant and anti‐neuronal apoptosis. Our study demonstrated that endogenous SIRT1 levels decreased and reached their lowest point at 3 days after ICH. The expressions of SIRT1 and 8‐OHdG showed a negative correlation after ICH. Baihui‐penetrating‐Qubin acupuncture could improve neurological deficits, brain edema, pathological injury, and reduce oxidative stress and neuronal apoptosis in ICH rats. Mechanistically, acupuncture activated the SIRT1/FOXO1signaling pathway, upregulating the protein expressions of SIRT1, FOXO1, SOD, GSH‐Px, CAT, and Bcl‐2, while downregulating the protein expressions of Ac‐FOXO1, MDA, 8‐OHdG, and Bax in the perihematomal area at 3 days after ICH. These beneficial effects of acupuncture were abolished by EX527. To our knowledge, this is the first study to use a SIRT1 inhibitor to demonstrate that the neuroprotective effects of scalp acupuncture and association with the regulation of oxidative stress and neuronal apoptosis in ICH involve modulation of SIRT1/FOXO1 signaling pathway.

SIRT1 is a nicotinamide adenine dinucleotide (NAD+)‐dependent class III histone deacetylase, which regulates a wide range of target genes by removing acetyl groups from histones and transcription factors, playing antioxidant and anti‐neuronal apoptosis effects (D'Angelo et al. 2021). High expression of SIRT1 has been observed in neurons of various brain regions, including the cerebral cortex, hippocampus, cerebellum, and hypothalamus, and it is implicated in neuronal plasticity and neuronal death (Tang et al. 2023; Zhang et al. 2016). In our study, we observed a time‐dependent decrease in SIRT1 expression, reaching its lowest levels in the perihematomal area at 3 days after ICH. Double immunofluorescence staining demonstrated that SIRT1 was predominantly present in the nucleus of neurons, and its expression was decreased in the perihematomal area at 3 days after ICH. These findings align with previous studies: (1) downregulation of SIRT1 protein levels has been reported in rats after ischemic stroke (Xu et al. 2020) and ICH (Deng et al. 2021); (2) SIRT1 is highly expressed in neurons but poorly expressed in microglia and astrocytes in the rat brain (Deng et al. 2021). 8‐OHdG is a marker of oxidative DNA damage. Oxidative stress leads to oxidative base modifications and double‐strand breaks in DNA, and the 8‐OHdG is a product of the interaction between DNA double‐strand breaks and hydroxyl radicals (Graille et al. 2020). In our study, we observed a negative correlation between the expressions of SIRT1 and 8‐OHdG. These findings suggest that SIRT1 may be involved in regulating oxidative stress after ICH.

Baihui‐penetrating‐Qubin acupuncture uses a needle to span three brain regions of the frontal, parietal, and temporal lobes, having a significant advantage in ICH treatment. Firstly, from the perspective of traditional Chinese medicine theory, GV20 and GB7 belong to scalp acupoints, which are the essential acupoints for improving neurological deficits in ICH patients (Liu et al. 2012; Zheng 2009). Secondly, based on modern medical research, acupuncture targeting GV20 could reduce brain edema, neuroinflammation, oxidative stress, apoptosis, and regulate cerebral blood flow, blood–brain barrier permeability, and promote nerve regeneration to protect ICH injury (Li et al. 2014). Additionally, acupuncture at GV20 and GB7 has been shown to recover cerebral structures associated with motor function in both hemispheres in stroke patients (Fang et al. 2012). Thirdly, penetrating needling therapy has significant advantage than basic acupuncture therapy. It involves convenient operation, a strong needling sensation (facilitating conduction), a minimal skin damage, and a more robust connection between meridians and collaterals (Seo et al. 2007). A published clinical trial highlighted that penetrating needling therapy on scalp acupoints can enhance hematoma absorption in acute ICH and improve neurological deficits in patients (Wang et al. 2016). Fourthly, considering the connection between neuroanatomical and functional aspects, the line connecting Baihui (GV20) and Qubin (GB7) acupoints is referred to as the top temporal posterior oblique line in the standard head acupoint line (Wang et al. 2010). Acupuncture in this area is particularly good at treating post‐stroke motor disorders and sensory abnormalities. Neurological deficits, brain edema, neuropathological damage are typical markers for the evaluation of secondary brain injury after ICH. In this study, Baihui‐penetrating‐Qubin acupuncture improved the neurological deficits and brain edema, as well as reduced the pathological injury and neuronal degeneration in the perihematomal area at 3 days after ICH. Baihui‐penetrating‐Qubin acupuncture attenuated oxidative stress injury and neuronal apoptosis after ICH, which was manifested in the decrease of neuronal apoptosis and 8‐OHdG in the perihematomal area, observed 3 days after ICH.

We further investigated the role of SIRT1 activation in antioxidant and anti‐neuronal apoptosis signaling pathways, following ICH and examined the neuroprotective mechanisms of acupuncture using the SIRT1‐specific inhibitor EX527. EX527, also known as Selisistat, is a potent and specific inhibitor of SIRT1. It functions by binding to the NAD+ and acetylated substrate‐binding sites, forming a stable ternary complex that effectively prevents the deacetylation process, thereby inhibiting SIRT1 activity (Gertz et al. 2013). This binding is competitive with the acetylated substrate and non‐competitive with NAD+, underscoring the specific targeting mechanism of EX527 (Scarano et al. 2024). The SIRT1/FOXO1 signaling pathway represents a unique mechanism for regulating oxidative stress and apoptosis. FOXO1, a member of the FOXO family, regulates the neurogenesis, differentiation, apoptosis, and oxidative stress (Santo and Paik 2018). The activity of the transcription factor FOXO1 is modulated by various upstream factors through translational modifications, including phosphorylation, methylation, glycosyl ubiquitination, ubiquitination, and acetylation (Hu et al. 2019). The SIRT1 promoter region has been reported to contain a forkhead‐like consistent binding site (FKHD‐L) that can bind to mammalian FOXO and influence its transcription levels (Kobayashi et al. 2005). Under oxidative stress, FOXO1 is deacetylated and activated by SIRT1, facilitating its translocation into the nucleus. Once in the nucleus, FOXO1 regulates the expression of genes related to antioxidation and cellular protection, including SOD, CAT, and GSH‐Px. These enzymes are essential for scavenging reactive ROS and mitigating oxidative damage (Hsu et al. 2010; X. S. Zhang et al. 2020). SOD, CAT, and GSH‐Px are common endogenous antioxidant enzymes, while MDA and 8‐OHdG are biomarkers of oxidative stress. MDA is the end product of lipid peroxidation, capable of reacting with proteins and nucleic acids to alter the structure and function of cells (J. Zhang et al. 2020). In our study, we found that Baihui‐penetrating‐Qubin acupuncture significantly upregulated the expressions of SIRT1, FOXO1, Bcl‐2, SOD, CAT, and GSH‐Px in the perihematomal area 3 days after ICH. Injection with EX527, a SIRT1 inhibitor, led to a significant decrease in the expressions of SIRT1 and its downstream molecules, including FOXO1, Bcl‐2, SOD, CAT, and GSH‐Px. Nonetheless, it increased the expressions of Ac‐FOXO1, Bax, and MDA. Hence, our findings suggest that Baihui‐penetrating‐Qubin acupuncture exerting antioxidant and anti‐neuronal apoptosis, at least in part, through the SIRT1/FOXO1 signaling pathway, improved neurological deficits after ICH.

We have conducted extensive foundational research across seven domains: behavioral, physiological, biochemical, pathological, immunological, molecular biology, and signal transduction (Guan et al. 2021; Kong et al. 2021; Li et al. 2020, 2021; Liu et al. 2017; H. Liu et al. 2021; P. Liu et al. 2021; Liu et al. 2018; Zhang et al. 2018; Zou et al. 2015) to elucidate the mechanisms by which Baihui‐penetrating‐Qubin acupuncture (hereinafter referred to as acupuncture) promotes neurological recovery following ICH. Acupuncture may regulate an extensive neuronal network that facilitates neuroplasticity after ICH. This includes: (1) inducing neurogenesis by regulating the Notch1/hes1 and Shh/Gli1 signaling pathways to promote neural stem cell proliferation and differentiation (Zhang et al. 2018; Zou et al. 2015); (2) promoting neurotransmitter release by enhancing the secretion of neurotrophic factors such as BDNF and GDNF, and growth factors like bFGF (Li et al. 2020); (3) inhibiting neuroinflammation by modulating the NF‐kB and Mincle/Syk signaling pathways (Liu et al. 2017, 2018); (4) regulating autophagy by initiating the autophagic self‐repair of neural cells post‐ICH (Guan et al. 2021; P. Liu et al. 2021); (5) modulating iron metabolism by activating the p62‐Keap1‐Nrf2 signaling pathway to regulate ferroptosis (Li et al. 2022); (6) inhibiting miR‐23a‐3p expression to alleviate neuronal cell death, neuroinflammation, and ferroptosis (Kong et al. 2021); and (7) inhibiting oxidative stress, as our current study demonstrates that acupuncture activates the SIRT1/FOXO1 signaling pathway to suppress oxidative stress and neuronal apoptosis following ICH.

Our study has demonstrated that acupuncture significantly impacts SIRT1 expression; however, the specific biological mechanisms underlying this effect require further elucidation. The literature suggests several potential pathways through which acupuncture may enhance SIRT1 expression and exert neuroprotective effects. Firstly, acupuncture may increase the NAD+/NADH ratio by reducing oxidative stress, which in turn activates SIRT1, contributing to neuroprotection by regulating oxidative stress pathways (Fangma et al. 2023). Secondly, acupuncture might enhance SIRT1 expression by activating AMP‐activated protein kinase (AMPK) (He et al. 2023), which raises NAD+ levels and boosts SIRT1 activity (Cantó et al. 2009). Additionally, electroacupuncture may stimulate the AMPK/SIRT1 pathway via TRPC1 activation (Geng et al. 2024). Thirdly, the metabolism of ketone bodies (KBs) plays a significant role in SIRT1 activation (Tozzi et al. 2022). As metabolic fuels, KBs directly interact with SIRT1, promoting fatty acid oxidation and energy metabolism conversion. They also increase NAD+ availability through metabolic pathways, providing essential cofactors for SIRT1 activation. Furthermore, KBs can influence the activity of histone deacetylases (HDACs) and work in conjunction with SIRT1 to regulate gene expression, thereby enhancing antioxidant and anti‐inflammatory responses. Experimental studies have shown that a ketogenic diet can significantly increase SIRT1 activity and improve mitochondrial function (Tozzi et al. 2022). However, the relationship between acupuncture and ketone body metabolism requires further investigation. While these mechanisms in ICH need additional validation, they provide a biological basis for understanding the neuroprotective effects of acupuncture and offer directions for future research.

## 5 Conclusion

In summary, this study showed that Baihui‐penetrating‐Qubin acupuncture could reduce oxidative stress and neuronal apoptosis through the SIRT1/FOXO1 signaling pathway, improving neurological deficits and neuropathological damage after ICH. These findings also indicate that Baihui‐penetrating‐Qubin acupuncture is an effective therapy for ICH, as well as targeting SIRT1 signaling, which could be a potential therapeutic strategy.

## Author Contributions

S.S.D designed the study and performed the experiments. S.S.D and M.Y.L drafted the manuscript. E.L.L and W.Z provided material support and essential advice in the study design. X.P.Y revised the manuscript. Y.N.K, X.H.D, L.Z, H.T.C, and W.H.D performed part of the experiments and analyzed the data. All authors reviewed and approved the final version of the manuscript.

## Acknowlegments

This work was supported by the National Natural Science Foundation of China (No.81473764), the Nature Science Foundation of Guangdong Province (2023A1515012779), and the Foundation of Shenzhen Science and Technology Innovation Committee (JCYJ20210324093213035). We would like to express our gratitude to EditSprings (https://www.editsprings.cn) for providing linguistic assistance.

## Conflicts of Interest

The authors declare no conflicts of interest.

### Peer Review

The peer review history for this article is available at https://publons.com/publon/10.1002/brb3.70095.

## Data Availability

The datasets used and/or analyzed during the current study are available from the corresponding author on reasonable request.
